# Context-Aware Emotion Recognition in the Wild Using Spatio-Temporal and Temporal-Pyramid Models

**DOI:** 10.3390/s21072344

**Published:** 2021-03-27

**Authors:** Nhu-Tai Do, Soo-Hyung Kim, Hyung-Jeong Yang, Guee-Sang Lee, Soonja Yeom

**Affiliations:** 1 Department of Artificial Intelligence Convergence, Chonnam National University, 77 Yongbong-ro, Gwangju 500-757, Korea; donhutai@gmail.com (N.-T.D.); hjyang@jnu.ac.kr (H.-J.Y.); gslee@jnu.ac.kr (G.-S.L.); 2 School of Technology, Environment and Design, University of Tasmania, Hobart, TAS 7001, Australia; Soonja.Yeom@utas.edu.au

**Keywords:** video emotion recognition, spatiotemporal, temporal-pyramid, best selection ensemble, facial emotion recognition

## Abstract

Emotion recognition plays an important role in human–computer interactions. Recent studies have focused on video emotion recognition in the wild and have run into difficulties related to occlusion, illumination, complex behavior over time, and auditory cues. State-of-the-art methods use multiple modalities, such as frame-level, spatiotemporal, and audio approaches. However, such methods have difficulties in exploiting long-term dependencies in temporal information, capturing contextual information, and integrating multi-modal information. In this paper, we introduce a multi-modal flexible system for video-based emotion recognition in the wild. Our system tracks and votes on significant faces corresponding to persons of interest in a video to classify seven basic emotions. The key contribution of this study is that it proposes the use of face feature extraction with context-aware and statistical information for emotion recognition. We also build two model architectures to effectively exploit long-term dependencies in temporal information with a temporal-pyramid model and a spatiotemporal model with “Conv2D+LSTM+3DCNN+Classify” architecture. Finally, we propose the best selection ensemble to improve the accuracy of multi-modal fusion. The best selection ensemble selects the best combination from spatiotemporal and temporal-pyramid models to achieve the best accuracy for classifying the seven basic emotions. In our experiment, we take benchmark measurement on the AFEW dataset with high accuracy.

## 1. Introduction

Emotional cues provide universal signals that enable human beings to communicate during the course of daily activities and are a significant component of social interactions. For example, people will use facial expressions such as a big smile to signal their happiness to others when they feel joyful. People also receive emotional cues (facial expressions, body gestures, tone of voice, etc.) from their social partners and combine them with their experiences to perceive emotions and make suitable decisions. In addition, emotion recognition, especially facial emotion recognition, has long been crucial in the human–computer interaction (HCI) field, as it helps computers efficiently interact with humans. Recently, several scientific studies have been conducted on facial emotion recognition (FER) in an attempt to develop methods based on new technologies in the computer vision and pattern recognition fields. This type of research has a wide range of applications, such as advertising, health monitoring, smart video surveillance, and development of intelligent robotic interfaces [1].

**Figure 1:**
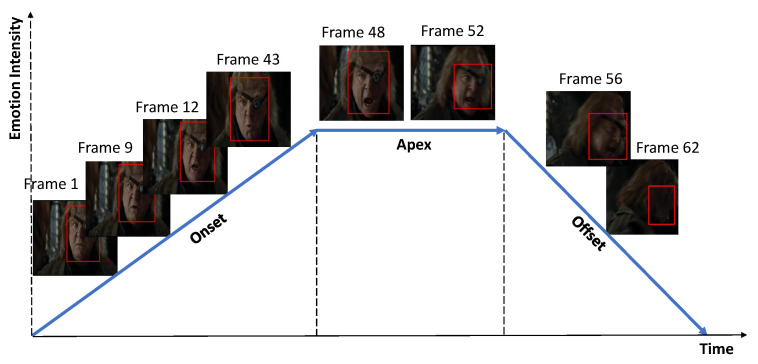
The three periods of facial expression representation are onset, apex, and offset. The duration of each varies, leading to unclear temporal borders. In addition, the appearance of spontaneous expressions leads to the presence of multiple apexes [6].

Emotion recognition on the basis of behavioral expressions presents numerous challenges due to the complex and dynamic properties of human emotional expressions. Human emotions change over time, are inherently multi-modal in nature, and differ in terms of such factors as physiology and language [2]. In addition, use of facial cues, which are considered the key aspect of emotional cues, still presents challenges owing to variations in such factors as head poses and lighting conditions [3]. Several factors, such as body expressions and tone of voice are also affected by noise in the environment and occlusion. In some cases, emotions cannot be interpreted without context [4]. In video-based emotion recognition, facial expression representation often includes three periods, onset, apex and offset [5,6], as shown in Figure 1. The lengths of the periods differ; the onset and offset periods tend to be shorter than the apex period. There are challenges regarding the unclear temporal border between periods, and spontaneous expressions lead to multiple apexes.

To address the above-mentioned challenges, both traditional and deep learning methods often focus on facial expressions that present changes in facial organs in response to emotional states, underlying intentions, and social interactions. Such methods attempt to determine facial regions of interest, represent changes in facial expressions, and divide emotions into six basic categories, namely, anger, disgust, fear, happiness, sadness, and surprise, as proposed by Ekman et al. [7].

In 2D image-based facial emotion recognition (2D FER), the main tasks focus on robust facial representation followed by classification. There are two approaches to feature representation, geometric- and appearance-based approaches. Geometric-based approaches represent facial expressions using geometric features of facial components (mouth, eyes, nose, etc.) in terms of shape, location, distance, and curvature [8,9,10]. Appearance-based approaches use local descriptors, image filters such as LBP [11], Gabor filters [12], PHOG [13], etc. to extract hand-crafted features for facial expression representation for traditional methods. In deep learning methods, feature representation is automatically extracted by convolutional neural networks (CNN) [14] that are trained on large-scale emotion recognition datasets such as RAF-DB [15] and AffectNet [16]. Geometric-based methods are often affected by noise and have difficulty showing small changes in facial details, while appearance-based methods are robust to noise and retain facial details. Deep learning models such as VGG-16 [17] and Resnet [18] demonstrate improved 2D FER performance [10,19].

In video-based emotion recognition, the main task focuses on efficiently exploiting spatiotemporal coherence to classify human emotion as well as integrating multiple modalities to improve overall performance. In the spatiotemporal approach, extensions of hand-crafted traditional features such as HOG, LBP and BoW are also proposed and applied using video-based emotion recognition methods such as 3D HOG [20], LBP-TOP [21], and Bag-Of-Word [22]. In addition, temporal models such as conditional random fields [23] and interval temporal Bayesian network [24] are used to exploit spatiotemporal relationships between different features. For deep learning-based methods, many works use CNNs for feature extraction followed by LSTM for exploiting spatiotemporal relations [25,26,27]. For the frame-level approach, every frame in a video clip is subjected to facial feature extraction, concatenated together by a statistical operator (mim, mean, and std) using pre-determined time steps and finally classified by deep learning models or traditional classification methods such as SVM [28,29].

Recently, many works have focused on video-based emotion recognition to address challenges in emotion recognition using the deep learning approach. Zhu et al. [30] used a hybrid attention cascade network to classify emotion recognition with a hybrid attention module for the fusion features of facial expressions. Shi et al. [31] proposed a self-attention module integrated with the spatial-temporal graph convolutional network for skeleton-based emotion recognition. Anvarjon et al. [32] proposed deep frequency features for speech emotion recognition.

However, video-based emotion recognition also presents some challenges under in-the-wild conditions, such as problems involving head pose, lighting conditions, and the complexity in the facial expression representation due to spontaneous expression. Context is key in emotion recognition. For instance, in a dark environment or when the face of interest is tiny, it is possible to recognize emotions based off our experiences with related elements such as parts of the scene, body gestures, things, and other people in the scene. In addition, a hierarchical structure in the emotion feature representation is necessary to deal with unclear emotion temporal borders.

In this study, we propose an overall system with face tracking and voting to select the main face for emotion recognition using two models based on spatiotemporal and temporal-pyramid architecture to efficiently improve emotion recognition. For face tracking and voting, we use a tracking-and-detection template with robust appearance features as well as motion features to suggest faces and people. Then, through a voting scheme based on probabilities, occurrences, and sizes, we choose the face and person of interest in the video clip.

In video-based emotion recognition, we first deal with in-the-wild conditions by integrating contextual features, facial emotion probability, and facial emotion features to construct a robust set of facial emotion features. For unclear temporal border and spontaneous expression problems, we propose a temporal-pyramid architecture to integrate face-context features by time steps based on statistical information. The hierarchical structure of facial-context feature integration improves the emotion evaluation results of our system. Moreover, we also propose a spatiotemporal model using “Conv2D+LSTM+3DCNN+Classify” architecture to exploit spatiotemporal coherence among face-context emotion features in 3D and 2D+T strategies. Finally, we suggest the best ensemble method to choose the best combination among models. Our experiment was conducted on the AFEW dataset [33] which is the dataset of the EmotiW Challenge 2019 [34]. We achieved good performance on the validation set and test set.

The contributions of this paper are as follows: (1) We integrate facial emotion features with scene context features to improve performance. (2) We propose spatiotemporal models to exploit spatiotemporal coherence among face-context features using 3D and 2D+T temporal strategies. In addition, we build a temporal-pyramid model to exploit the hierarchical structure of overall face-context emotion features by statistical operator. (3) Our proposed system achieved good performance on a validation set taken from the AFEW dataset [33].

This paper is organized into seven sections. In Section 2, we briefly summarize related works. We describe our proposed idea in Section 3. We discuss the network architectures in Section 4 and the best selection ensemble method in Section 5. Our experiments are shown in Section 6. Finally, the conclusions are outlined in Section 7.

## 2. Related Works

### 2.1. Image-Based Facial Expression Recognition

Emotion recognition plays a fundamental role in human–computer interactions (HCIs). It is used to automatically recognize emotions for a wide range of applications, such as customer marketing, health monitoring, and emotionally intelligent robotic interfaces. Emotion recognition remains a challenging task due to the complex and dynamic properties of emotions, their tendency to change over time, the fact that they are often mixed with other factors, and their inherently multi-modal nature in terms of behavior, physiology, and language.

To recognize emotion expression, the face is one of the most important visual cues. Facial expression recognition (FER) exploits the facial feature representation of static images [11] in the spatial domain. Traditional methods use handcrafted features such as local binary patterns (LBPs), speeded-up robust features (SURF), and scale-invariant feature transform (SIFT), to classify emotions. Recently, with the success of deep learning in computer vision tasks, FER problems raise the new challenge for classifying emotions under in-the-wild environments despite occlusions, illumination differences, etc. Many 2D FER image datasets such as AffectNet [16], RAF-DB [15], etc. have been published to promote technological development and fulfill the requirement for large-scale and real-world datasets.

### 2.2. Video-Based Emotion Recognition

From still images to video, emotion recognition presents many serious challenges; these involve, for example, behavioral complexities, environmental effects, and temporal changes in the video channel, as well as acoustic and language differences in the audio channel. To provide a baseline for video emotion recognition in the wild, the AFEW dataset [33] was built from many movies and TV shows. Emotions are classified into seven categories (anger, disgust, fear, happiness, neutrality, sadness, and surprise) under uncontrolled environments such as outdoor/indoor scenes, illumination changes, occlusions, and spontaneous expression. From 2013 to 2018, the emotion recognition research community made great strides through the EmotiW Challenge [34] on the basis of the AFEW dataset [33].

Because human emotions are almost always displayed on the face by movements of facial muscles, many studies have focused on facial representations in attempts to exploit the spatial and temporal information contained in a video. There are three main approaches to this problem: geometry, video-level, and frame-level approaches.

For the geometry approach, Liu et al. [26] computed 3D landmarks, normalized these landmarks and extracted features using Euclidean distances. They proposed the Landmark Euclidean Distance network. Kim et al. [27] proposed the CNN-LSTM network to classify emotions through sequential 2D landmark features.

For the spatiotemporal approach, Liu et al. [26] used the VGG Face network to extract facial features and then used these facial features to classify emotions. They showed an accuracy of 43.07% on the validation set. Lu et al. [25] proposed VGG-Face+BLSTM [35] for the spatiotemporal network using the VGG-Face network fine-tuned on facial expression images from video clips. This model showed an accuracy of 53.91%.

Finally, the main idea of the frame-level approach is to merge emotion features in every frame using an aggregation function (min, max, std, etc.). It addresses the invariance of the number of video frames. Bargal et al. [29] used facial emotion recognition networks to extract facial features and concatenated the results.For all frames, they used the statistical encoding module (STAT) to merge all frame-level features by min, max, variance, and average. They showed a high accuracy of 58.9% on the validation set. Knyazev et al. [28] later updated the STAT* module by scaling and normalization.

We realize that weak points exist in the above works that make use of the spatiotemporal, frame-level, and audio modalities. For instance, the spatiotemporal networks do not integrate 3DCNN [36] and BiLSTM [35] to find strong correlations between the spatial information in the data cube. Moreover, it would be better to use online fine-tuning in the video training process instead of offline feature extraction.

For the frame-level approach, STAT encoding does not utilize temporal information between the frame-level features. In addition, the frame-level features need to add more contextual information such as action information and scene information. The audio approach only uses one type of acoustic feature for emotion classification.

## 3. Proposed Idea

In this section, we define the problem that we wish to address and give a brief overview of our video emotion recognition system. Next, we explain our proposed method in detail, including the tracking and voting modules and method of face context feature extraction. The details of the model are discussed in the next section.

### 3.1. Problem Definition

In this study, the input is a video clip $V=\left\{S,A\right\}$ lasting 5 min or less consisting of a scene sequence S and audio stream A. Certain cues play an important role in human emotion recognition, such as facial expression, body gestures, and tone of voice. In the scope of our work, we mainly focus on visual cues that are important to the perception of human feelings. Face tracking F, along with the corresponding person tracking P comprising body and scene information, are the most important cues to solve this problem. Our objective is to effectively locate the significant face $\bar{F}$ and corresponding person $\bar{P}$ from the scene sequence S. From there, we use the face and person image sequences $S_{\bar{F}}$ and $S_{\bar{P}}$ to classify the emotion $c_{i}\in C=\left[0,6\right]$ as one of seven basic emotions, namely, anger, disgust, fear, happiness, neutrality, sadness, and surprise.

**Figure 2:**
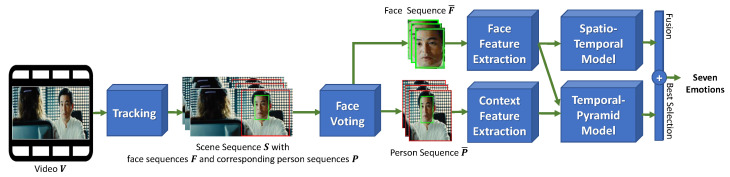
Overview of the proposed system for video emotion recognition in the wild.

Let $c_{fj}^{t}\overset{\Delta }{=}\left(x_{fj}^{t},y_{fj}^{t},w_{fj}^{t},h_{fj}^{t}\right)\in R^{4}$ and $c_{pj}^{t}\overset{\Delta }{=}\left(x_{pj}^{t},y_{pj}^{t},w_{pj}^{t},h_{pj}^{t}\right)\in R^{4}$ be the location of the *j*th face and person at time *t* in a scene sequence $S\in R^{W\times H\times T}$, respectively, and the tracking indices $g_{j}^{t}\in R$ calculated using the tracking module shown in Figure 2, where $\left(x_{fj}^{t},y_{fj}^{t}\right)/\left(x_{pj}^{t},y_{pj}^{t}\right)$ is the face/person center, $\left(w_{fj}^{t},h_{fj}^{t}\right)/\left(w_{pj}^{t},h_{pj}^{t}\right)$ is the face/person size, W×H is the size of a scene, and *T* is the length of scene sequence S. The scene sequence S then contains the face tracking F and person tracking P information, defined as follows:

(1) $$\begin{array}{c} \begin{array}{cc} F & ={\left\{f_{i}\right\}}^{i=1\cdots N_{f}}\;\mathrm{and}\;P={\left\{p_{i}\right\}}^{i=1\cdots N_{f}},\\ f_{i} & ={\left\{c_{fj_{k}}^{t_{k}}|t_{k}<t_{k+1}\wedge g_{j_{k}}^{t_{k}}=i\right\}}^{k=1\cdots M_{i}}\;\mathrm{and}\;\\ p_{i} & ={\left\{c_{pj_{k}}^{t_{k}}|t_{k}<t_{k+1}\wedge g_{j_{k}}^{t_{k}}=i\right\}}^{k=1\cdots M_{i}} \end{array} \end{array}$$

where $N_{f}$ is the number of tracked faces and persons and $f_{i}$/$p_{i}$ is the *i*th tracked face/person that contains the locations of the face/person in chronological order ($t_{k}<t_{k+1}$) and which has a length of $M_{i}$ and the same tracking index ($g_{j_{k}}^{t_{k}}=i$).

We also denote $S_{f_{i}}$ and $S_{p_{i}}$ as, respectively, the image sequences of a tracked face and person, $f_{i}$ and $p_{i}$, extracted from the scene sequence S. The emotional expression in the video V is mostly affected by the most significant face $\bar{F}$, which appears more often and is larger than the other faces, and the corresponding person $\bar{P}$, defined as follows:

(2) $$\begin{array}{c} \begin{array}{cc} \bar{F} & =mode\left\{F\right\}\\ \bar{P} & =select_{g_{\bar{P}}=g_{\bar{F}}}\left\{P\right\} \end{array} \end{array}$$

where $g_{\bar{P}}$ and $g_{\bar{F}}$ are the tracking indices of $\bar{P}$ and $\bar{F}$, respectively.

The goal of our method is to classify the image sequences $S_{\bar{F}}$ and $S_{\bar{P}}$ of the dominant tracked face $\bar{F}$ and corresponding person $\bar{P}$ to classify what kind of emotions exist in the video V. The classification result is denoted by a classification label $c\in \left[0,6\right]$ corresponding to the seven basic emotions anger, disgust, fear, happiness, neutrality, sadness, and surprise.

### 3.2. Proposed System

An overview of our proposed system is shown in Figure 2. The system attempts to classify a video clip in the wild according to seven categorical emotions, namely anger, disgust, fear, happiness, neutrality, sadness, and surprise.

The key to this study is context-aware emotion recognition in video clips. The expression of the key face in a video clip signifies the emotion that the system will apply to that clip. The contextual features from the person region are used to improve the performance of the system when the key face is small and/or occluded. Our proposed model exploits the context-aware feature map to classify emotions into seven basic categories.

First, from an input video clip, our system effectively locates the most important tracked face $\bar{F}$ and corresponding tracked person $\bar{P}$ using the Tracking and FaceVoting module. These are considered the most significant characteristics to help our system classify emotional expression.

Second, the face context feature map is extracted from the significant face $\bar{F}$ and person $\bar{P}$ using the face feature extraction and context feature extraction models. The face feature extraction model is based on conventional models and uses pre-trained weights based on the AffectNet [16] and RAF-DB [15] datasets. The context feature extraction model is VGG16 [17], with pre-trained weights from ImageNet.

The context spatiotemporal LSTM-3DCNN model uses LSTM [37] or 3DCNN [36] to exploit the spatiotemporal correlation of the face context feature map and fine-tune the face feature extraction model. Its scheme is “FaceContext+LSTM+Conv3D+Classification” and it helps our system learn the feature map more deeply.

Moreover, we propose the context temporal-pyramid model based on the temporal-pyramid scheme instead of LSTM and 3DCNN. The face context feature map can be enhanced by the temporal-pyramid scheme as well as statistical operators (mean, max, and min). It exploits the long-term dependencies in all time-steps from the face context feature map. Our system applies categorical cross-entropy loss for training on the seven basic emotion classes for every video emotion model.

Finally, we fuse the classification features from all models to achieve the best accuracy in emotion classification. We propose the best selection ensemble and compare it to average fusion and join fine-tuning fusion [10]. The best selection ensemble finds the best combination of models by the heuristic principle when giving a first specific model. It attempts to find an unused model to help the current combination achieve the best accuracy with a smaller number of models to prevent over-fitting.

### 3.3. Face and Person Tracking

For the tracking module, we propose a tracking algorithm based on a tracking-by-detection scheme [38] and Hungarian matching method [39] to return the tracked faces F along with the corresponding tracked persons P from the scene sequence S.

#### 3.3.1. Tracking Database of Tracked Faces and Persons

It is assumed that there are tracked faces $F={\left\{f_{i}\right\}}^{i=1\cdots N_{t}}$ and corresponding tracked persons $P={\left\{p_{i}\right\}}^{i=1\cdots N_{t}}$ at the time *t*, where $N_{t}$ is the number of tracked faces and persons, and $f_{i}$ (or $p_{i}$) is the location sequence of a tracked face (or person) as defined in Equation (1). Let $D={\left\{d_{i}\right\}}^{i=1\cdots N_{t}}$ be the tracking database containing appearance and motion observations.

Our algorithm uses the HSV color histogram and the face features to record appearance observations. The last face size and location of a tracked face record motion observations. Each element $d_{i}=\left(d_{i}^{hsv},d_{i}^{enc},d_{i}^{pos},d_{i}^{size}\right)\in D$ is calculated as follows:

(3) $$\begin{array}{c} \begin{array}{cc} d_{i}^{hsv} & ={\left\{H_{hsv}\left(S_{f_{i}^{j}}\right)\right\}}^{j=M_{i}-k+1\cdots M_{i}}\\ d_{i}^{enc} & ={\left\{G_{vggface2}\left(S_{f_{i}^{j}}\right)\right\}}^{j=M_{i}-k+1\cdots M_{i}}\\ d_{i}^{pos} & ={\left(x,y\right)}_{f_{i}^{M_{i}}}\;\mathrm{and}\;d_{i}^{size}={\left(w,h\right)}_{f_{i}^{M_{i}}} \end{array} \end{array}$$

where $d_{i}^{hsv}$ is the HSV color histograms of the last k-face images $\left\{S_{f_{i}^{j}}\right\}$ for the tracked face $f_{i}$; $M_{i}$ is the number of faces in $f_{i}$; and the operator $H\left(.\right)$ is used to generate 100 bin values of a 2D histogram using the H and S channels for color, and 20 bin values of a 1D histogram using the V channel for brightness, as mentioned in [40]. $d_{i}^{enc}$ is the face encoding features of the last k-face images, which is extracted from the model G that uses pre-trained weights from VGGFace2 [41]. $d_{i}^{pos}$ and $d_{i}^{size}$ are respectively the last position and size of the tracked face $f_{i}$.

#### 3.3.2. Face and Person Candidates

For every scene $s_{t}\in S$, our algorithm uses Tiny Face Detector [42] to extract face candidates. This is is a robust detector that finds small faces with high efficiency. We also use SSD detection [43] trained on the VOC dataset to detect person candidates. For every face candidate, we find the person candidate that yields the smallest intersect over union (IoU) score. If this is not possible, the whole scene is used as the person region.

Let $\forall c_{fj}^{t}=\left(x_{fj}^{t},y_{fj}^{t},w_{fj}^{t},h_{fj}^{t}\right)\in C_{F}$ and $\forall c_{pj}^{t}=\left(x_{pj}^{t},y_{pj}^{t},w_{pj}^{t},h_{p,j}^{t}\right)\in C_{P}$ be, respectively, the face and person candidates in the scene $s_{t}$, where $\left(x_{fj}^{t},y_{fj}^{t}\right)$/$\left(x_{pj}^{t},y_{pj}^{t}\right)$ is the face/person center, and $\left(w_{fj}^{t},h_{fj}^{t}\right)$/$\left(w_{pj}^{t},h_{pj}^{t}\right)$ is the face/person size. We need to extract appearance and motion observations of the face candidates. Let $O_{F}=\left\{o_{j}\right\}$ be the appearance and motion observations of face candidates $C_{F}$; then, every element $o_{j}=\left(o_{j}^{hsv},o_{j}^{enc},o_{j}^{pos},o_{j}^{size}\right)$ is computed as follows:

(4) $$\begin{array}{c} \begin{array}{cc} o_{j}^{hsv} & =H_{hsv}\left(S_{c_{fj}^{t}}\right)\;\mathrm{and}\;o_{j}^{enc}=G_{vggface2}\left(S_{c_{fj}^{t}}\right)\\ o_{j}^{pos} & ={\left(x,y\right)}_{c_{fj}^{t}}\;\mathrm{and}\;o_{j}^{size}={\left(w,h\right)}_{c_{fj}^{t}} \end{array} \end{array}$$

where $S_{c_{fj}^{t}}$ is the corresponding image of $c_{fj}^{t}$, the operator $H\left(.\right)$ is used to extract the HSV color histogram, and the pre-trained VGGFace2 model G is used to compute the face-encoding features.

#### 3.3.3. Face and Person Matching

Let $M^{v}$ be the cost matrix of the observation $v\in \left\{hsv,enc,pos,size\right\}$ between the face candidates $C_{F}$ and the tracked faces F. We use a Euclidean distance operator $E\left(.\right)$ to calculate every element $M_{ij}^{v}\in M^{v}$ as follows:

(5) $$\begin{array}{c} M_{ij}^{v}=\left\{\begin{array}{cc} E\left({\bar{d}}_{i}^{v},o_{j}^{v}\right) & if\;E\left({\bar{d}}_{i}^{v},o_{j}^{v}\right)\leq T^{v}\\ \infty & otherwise \end{array}\right. \end{array}$$

where the operator ${\bar{d}}_{i}^{v}$ is the mean of $d_{i}^{v}$, $T^{v}$ is the valid threshold of observation *v* (determined experimentally), *i* is the face index in F, and *j* is the candidate index in $C_{F}$ and $C_{P}$.

The total cost matrix M is the weighted sum of $\forall M^{v}$ with every element $M_{ij}$ calculated as follows:

(6) $$\begin{array}{c} M_{ij}=\sum_{v}w_{v}\frac{M_{ij}^{v}}{\sum_{\notin \infty }M^{v}} \end{array}$$

where $w_{v}$ is the weighted term of the observation *v* and $\sum_{\notin \infty }$ is the sum of elements other than *∞*.

Our algorithm uses the Hungarian matching method [39] to find the optimal solution for which each tracking candidate $c_{fj}^{t}$ (or $c_{pj}^{t}$) is assigned to at most one tracking object $f_{i}$ (or $p_{i}$) and each tracking object $f_{i}$ (or $p_{i}$) is assigned to at most one tracking candidate $c_{fj}^{t}$ (or $c_{pj}^{t}$) as follows:

(7) $$\begin{array}{c} \sum_{i}\sum_{j}M_{ij}X_{ij}\to min \end{array}$$

where X is a Boolean matrix with $X_{ij}=1$ if the tracking candidate $c_{fj}^{t}$ (or $c_{pj}^{t}$) is assigned to the tracking object $f_{i}$ (or $p_{i}$).

Then, we compute tracking indices $\left\{g_{j}^{t}\right\}$ to assign the *j*th tracking candidate $c_{fj}^{t}$ (or $c_{pj}^{t}$) to the tracked objects F (or P) as follows:

(8) $$\begin{array}{c} g_{j}^{t}=\left\{\begin{array}{cc} i & if\;X_{ij}=1\wedge \forall v,M_{ij}^{v}\neq \infty \\ \infty & otherwise \end{array}\right. \end{array}$$

#### 3.3.4. Face and Person Update

For $g_{j}^{t}=i$, the tracking candidate $c_{fj}^{t}$ (or $c_{pj}^{t}$) is assigned to the tracked object $f_{i}$ (or $p_{i}$) as follows:

(9) $$\begin{array}{c} \begin{array}{cc} f_{i} & =f_{i}⊕c_{j}\\ d_{i}^{v} & =d_{i}^{v}⊕o_{j}^{v}\;,\;v\in \left\{hsv,enc\right\}\\ d_{i}^{v} & =o_{j}^{v}\;,\;v\in \left\{pos,size\right\} \end{array} \end{array}$$

where the operator ⊕ is used to insert an element into the last position of an array.

Otherwise, for $g_{j}^{t}=\infty$, the candidate $c_{fj}^{t}$ (or $c_{pj}^{t}$) is a new tracking object to be inserted into the set of tracked objects F (or P) as follows:

(10) $$\begin{array}{c} \begin{array}{cc} F & =F⊕\left\{c_{j}\right\}\\ D & =D⊕\left\{o_{j}^{v}\right\},v\in \left\{hsv,enc,pos,size\right\} \end{array} \end{array}$$

### 3.4. Face Voting

For the FaceVoting module, the system votes on the most significant face that has the largest influence on human emotional perception. Therefore, the inputs are the tracked faces F and tracked persons P. The outputs are the most significant tracked face $\bar{F}$ and the corresponding person $\bar{P}$, which are used in the emotion classification.

The most important tracked face is the face that occurs more often and more clearly than the other tracked faces. It is assessed through frequency of occurrence, face size, and face probability. Given the tracked faces $F={\left\{f_{i}\right\}}^{i=1\cdots M_{i}}$ and persons $P={\left\{p_{i}\right\}}^{i=1\cdots M_{i}}$, the weighted terms of frequency of occurrence, face size, and face probability of each tracked face $f_{i}$ and tracked person $p_{i}$ are computed as follows:

(11) $$\begin{array}{c} \begin{array}{cc} w_{freq}^{i} & =\frac{M_{i}}{T}\\ w_{size}^{i} & =\frac{\sum_{j=1}^{M_{i}}w_{j}^{i}\times h_{j}^{i}}{M_{i}\times W\times H}\\ w_{prob}^{i} & =\frac{\sum_{j=1}^{M_{i}}p_{j}^{i}}{M_{i}} \end{array} \end{array}$$

where $\left(W,H\right)$ and *T* are, respectively, the size and length of the scene sequence S and $\left(w_{j}^{i},h_{j}^{i}\right)$ and $p_{j}^{i}$ are, respectively, the size and detection probability of the *j*th face in the tracked face $f_{i}$.

The weighted term of each tracked face $f_{i}$ and tracked person $p_{i}$ is calculated as follows:

(12) $$w^{i}=c_{freq}w_{freq}^{i}+c_{size}w_{size}^{i}+c_{prob}w_{prob}^{i}$$

where $c_{x\in \left\{freq,size,prob\right\}}$ is a constant term that is used to adjust the priority of frequency of occurrence, face size, and face probability features in the face voting process.

The significant tracked faces $\bar{F}$ and corresponding tracked persons $\bar{P}$ have a weight that reaches a maximum value:

(13) $$\begin{array}{c} \begin{array}{cc} i_{max} & =\underset{i\in \left[1\cdots M_{i}\right]}{\arg\max}\;w^{i}\\ \bar{F} & =f_{i_{max}}\\ \bar{P} & =p_{i_{max}} \end{array} \end{array}$$

From there, we extract the face images $S_{\bar{F}}$ and person images $S_{\bar{P}}$ based on tracked face $\bar{F}$ and tracked person $\bar{P}$, respectively.

### 3.5. Face and Context Feature Extraction

**Figure 3:**
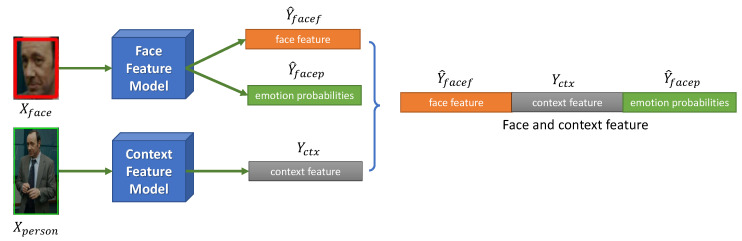
Face and Context Feature Extraction.

The Face and Context Feature Extraction module produces face and context features and probabilities for each of the seven emotions from the face and person regions using the face and context feature extraction models shown in Figure 3.

Let $M_{face}$ be the face feature model which is built on conventional base networks such as Resnet [18], SEnet [44], Xception [45], Nasnet mobile [46], Densenet [47], Inception Resnet [48], VGG Face 1 [49], VGG Face 2 [41], and ImageNet [50]. The model receives a face image $X_{face}$ and returns prediction emotion probabilities ${\hat{Y}}_{facep}\in R^{7}$ and feature vector ${\hat{Y}}_{facef}\in R^{K}$ as follows:

(14) $$\begin{array}{c} \begin{array}{cc} {\hat{Y}}_{facep},{\hat{Y}}_{facef} & =M_{face}\left(X_{face}\right) \end{array} \end{array}$$

where *K* is the feature size and ${\hat{Y}}_{facep}$ is the one-hot encoding vector used to determine the emotion label *c* by $c=\arg\max{\hat{Y}}_{facep}$.

In this study, we trained $M_{face}$ on the AffectNet dataset [16] and fine-tuned it on the RAF-DB dataset [15] with category cross-entropy (CCE) loss as follows:

(15) $$\begin{array}{c} \begin{array}{cc} L_{CCE} & =-\sum_{c\in C}Y_{facep}\log{\hat{Y}}_{facep} \end{array} \end{array}$$

where *c* is the emotion label in the set of seven basic emotions C.

Similarly, let $M_{ctx}$ be the context feature model, which extracts the context feature vector $Y_{ctx}$ from the person image $X_{person}$ as follows:

(16) $$\begin{array}{c} \begin{array}{cc} Y_{ctx} & =M_{ctx}\left(X_{person}\right) \end{array} \end{array}$$

where the context feature extraction model $M_{ctx}$ is built on the VGG16 model [17] with weights pre-trained on ImageNet.

Formally, we want the face feature model $M_{face}$ and the context model $M_{ctx}$ to follow the following distribution:

(17) $$\begin{array}{c} \begin{array}{cc} p\left(c|X_{face},X_{person}\right) & =p\left(c|{\hat{Y}}_{facef},{\hat{Y}}_{facep},Y_{ctx}\right) \end{array} \end{array}$$

The context around a person’s region is used to improve the performance of our model when the tracked face is very small or occluded. By extracting the feature vector with a model trained on ImageNet, we exploit the image diversity in ImageNet, and integrate this information into the face feature vector to identify correlations among the face and context characteristics and the emotion probability vector.

## 4. Network Architectures

### 4.1. Context Spatiotemporal LSTM-3DCNN Model

**Figure 4:**
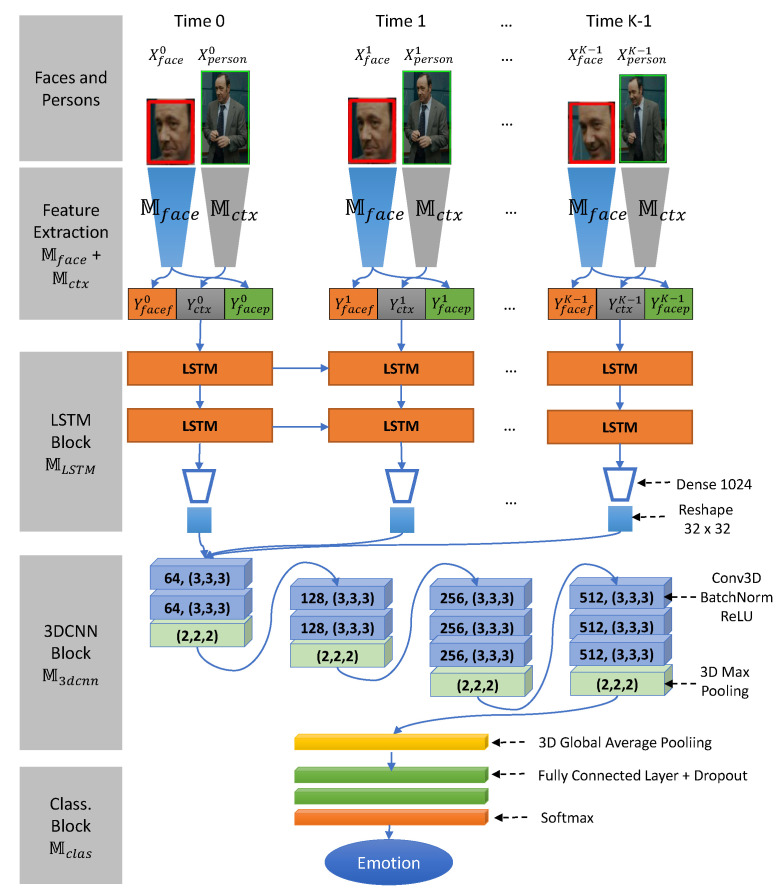
Context Spatio-Temporal LSTM-3DCNN Model.

**Overview**. The context spatiotemporal LSTM-3DCNN model shown in Figure 4 incorporates the face, context feature blocks $M_{face}$ and $M_{ctx}$, the LSTM block $M_{LSTM}$, the 3DCNN block $M_{3dcnn}$, and the classification block $M_{clas}$. Our proposed model uses the face and context feature blocks $M_{face}$ and $M_{ctx}$ to extract the face and context feature vectors. Use of the context feature vector helps to improve the accuracy of our model in difficult cases such as those with occluded face, small face, etc. Next, the LSTM block $M_{LSTM}$ exploits the temporal correlation among the feature vectors and normalizes the information to a fixed-length spatiotemporal feature map where the first axis is the temporal dimension and the second and third axes are the spatial dimension. The 3DCNN block $M_{3dcnn}$ learns spatiotemporal information from the spatiotemporal feature map to produce the high-level emotional features. From there, the classification block $M_{clas}$ classifies the emotion as one of the seven basic categories.

The context feature vectors play an important role in performance improvement. It deals with the difficulties in emotion recognition when the faces are occluded and small. Moreover, it integrates contextual features with body posture, visual scene, social situations, etc. to explain human emotion instead of using only facial cues in emotion recognition.

**Implementation Details**. Given the significant tracked face $S_{\bar{F}}$ and corresponding person $S_{\bar{P}}$ in the input image sequences, the module applies random temporal sampling to transform the input image sequences into sequences with a fixed length of *K* as follows:

(18) $$\begin{array}{c} \begin{array}{cc} {\left\{X_{face}^{t}\right\}}_{t=1}^{K} & =TemporalSampling\left(S_{\bar{F}}\right)\\ {\left\{X_{person}^{t}\right\}}_{t=1}^{K} & =TemporalSampling\left(S_{\bar{P}}\right) \end{array} \end{array}$$

where *K* is the size of the sampling operator with a value of 32.

The network uses the face and context feature blocks $M_{face}$ and $M_{ctx}$ to transform every input face image $X_{face}^{t}$ and person image $X_{person}^{t}$ at time step $t=\bar{1,K}$ in the input sequences. The outputs return the face probability vector $Y_{facep}^{t}$, face feature vector $Y_{facef}^{t}$, and context feature vector $Y_{ctx}^{t}$:

(19) $$\begin{array}{c} \begin{array}{cc} Y_{facep}^{t},Y_{facef}^{t} & =M_{face}\left(X_{face}^{t}\right)\\ Y_{ctx}^{t} & =M_{ctx}\left(X_{person}^{t}\right) \end{array} \end{array}$$

Finally, they are combined to form the overall face context feature vector $Y_{face\_ctx}^{t}$ as follows:

(20) $$\begin{array}{c} \begin{array}{cc} Y_{face\_ctx}^{t} & =concat\left(Y_{facep}^{t},Y_{facef}^{t},Y_{ctx}^{t}\right)\\ & =concat\left(M_{face}\left(X_{face}^{t}\right),M_{ctx}\left(X_{person}^{t}\right)\right) \end{array} \end{array}$$

where the concat operator is used to combine feature vectors.

We freeze the first layers of $M_{face}$ with the exception of the end layers, which have roles in feature extraction and emotion classification. This helps the face feature model $M_{face}$ not only transfer knowledge from the model pre-trained on large-scale image emotion recognition datasets [15,16] but also to be fine-tuned again at frame level on the video emotion dataset [33]. For $M_{ctx}$, we freeze all layers and only extract the context feature that is learned from the model that is pre-trained on the large-scale ImageNet dataset [50].

To exploit the long-term dependencies, the LSTM block $M_{LSTM}$ consists of stacked LSTM layers where each LSTM memory cell at layer i computes the hidden and state vectors $h_{i}^{t}$, $c_{i}^{t}$ from the current face context feature $Y_{face\_ctx}^{t}$ (for layer 0) or the hidden vector $h_{i-1}^{t}$ (for layer i > 0), and the hidden and cell states after the previous LSTM memory cell $h_{i}^{t-1}$, $c_{i}^{t-1}$:

(21) $$\begin{array}{c} \begin{array}{cc} h_{i}^{t},c_{i}^{t} & =\left\{\begin{array}{cc} LSTM\left(Y_{face\_ctx}^{t},h_{0}^{t-1},c_{0}^{t-1}\right), & i=0\\ LSTM\left(h_{i-1}^{t},h_{i}^{t-1},c_{i}^{t-1}\right), & 0<i<L \end{array}\right. \end{array} \end{array}$$

where *L* is the number of LSTM layers in $M_{LSTM}$. In this study, we chose L=2 by experiment.

Next, we use the Dense and Reshape layers to normalize every hidden state vector $h_{L-1}^{t}$ at the last LSTM layer to a specific length and produce the spatiotemporal feature map $Y_{lstm}\in R^{K\times S\times S}$ of the face and context feature vectors $\left\{Y_{face\_ctx}^{t}\right\}$ as follows:

(22) $$\begin{array}{c} \begin{array}{cc} Y_{lstm} & =\left\{Reshape_{S\times S}\left(Dense_{L}\left(h_{L-1}^{t}\right)\right)\right\} \end{array} \end{array}$$

where L=S×S, and $\left(S\times S\right)$ are the fixed-length and (width, height) used to normalize and reshape the hidden state vector, respectively, and *K* is the number of time-steps.

To perform a deeper analysis of the spatiotemporal feature map $Y_{lstm}$ in the temporal domain and ensure spatial coherence of the feature domain, the 3DCNN block $M_{3dcnn}$ is used to produce the emotional high-level feature $Y_{3dcnn}$ from $Y_{lstm}$ as folows:

(23) $$\begin{array}{c} \begin{array}{cc} Y_{3dcnn} & =M_{3dcnn}\left(Y_{lstm}\right) \end{array} \end{array}$$

where $M_{3dcnn}$ consists of four 3D convolutional blocks and a global average pooling layer. Every 3D convolutional block has 3D convolutional layers, followed by a batch normalization layer, and a rectified linear unit (ReLU), along with a 3D max pooling layer, at the end. The number of 3D convolutional layers and the kernel size of each one are, respectively: (2, 64), (2, 128), (3, 256), and (4, 512). All 3D convolutional layers use 3×3×3 filters and a padding of 1. The 3D max pooling layers have a size of 2×2×2.

Lastly, $M_{clas}$ receives the emotion feature $Y_{3dcnn}$ and classifies it into the seven basic emotions. $M_{clas}$ comprises two fully-connected layers followed by ReLU layers and dropout layers. At the end of the block, a softmax layer is used to output the emotion probability vector $Y_{emotion}$ as follows:

(24) $$\begin{array}{c} \begin{array}{cc} Y_{emotion} & =M_{clas}\left(Y_{3dcnn}\right) \end{array} \end{array}$$

Finally, we use categorical cross-entropy loss for emotion classification as follows:

(25) $$\begin{array}{c} \begin{array}{cc} CCE\left(Y_{gt\_emotion},Y_{emotion}\right) & =-\sum_{i=1}^{C}Y_{i,gt\_emotion}\log Y_{i,emotion} \end{array} \end{array}$$

where $Y_{gt\_emotion}$ is the ground-truth; $Y_{emotion}$ is the prediction result of the model; and *C* is the number of emotion labels.

### 4.2. Context Temporal-Pyramid Model

**Figure 5:**
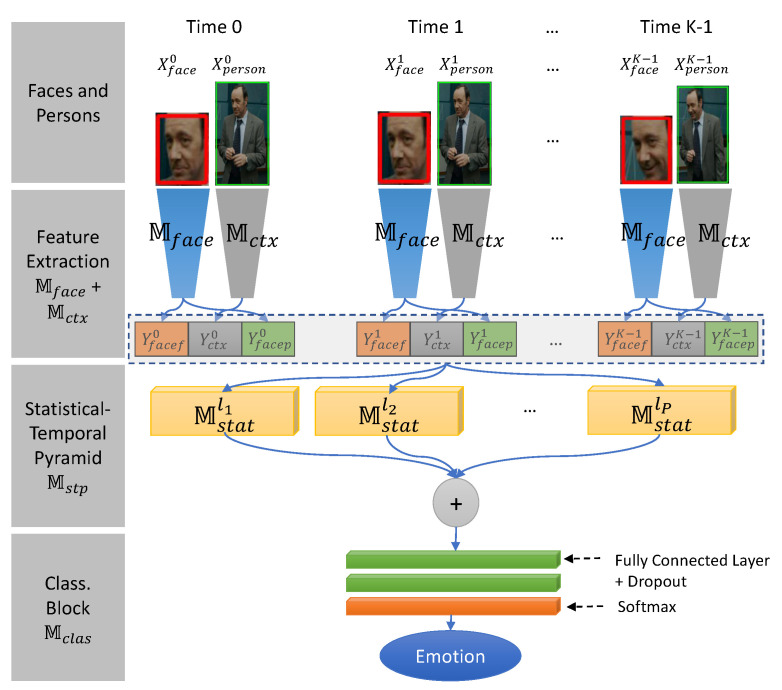
Context Temporal-Pyramid Model.

**Overview**. The context temporal-pyramid model illustrated in Figure 5 comprises the face and context blocks $M_{face}$ and $M_{ctx}$, the temporal-pyramid block $M_{stp}$, and the classification block $M_{clas}$. The model has some similarities to the context spatiotemporal model in that it uses $M_{face}$ and $M_{ctx}$ for face context feature extraction and $M_{clas}$ for emotion classification. However, the model exploits the face context features during all time steps in long-term temporal dependencies. The temporal-pyramid block $M_{stp}$ provides all face context features from the feature extraction block to the statistical aggregation $M_{stat}^{k=l_{1},l_{2},\ldots ,l_{P}}$ models where *P* is the number of statistical aggregation models. Each $M_{stat}^{k}$ builds the temporal pyramid features at level k. It will divide the time steps into $2^{k}$ feature sub-sequences and aggregate the face and context features using the mean operator and face probabilities by max, mean, and min operators. From there, all temporal pyramid features at all pyramid levels are combined into the context temporal pyramid feature to exploit the long-term dependencies of the face context features in all time-steps. Finally, emotion classification is done by $M_{clas}$.

**Implementation Details**. The context temporal-pyramid model uses all faces and persons in $S_{\bar{F}}$ and $S_{\bar{P}}$ to extract face context features ${\left\{Y_{face_{c}tx}^{t}=\left(Y_{facep}^{t},Y_{facef}^{t},Y_{ctx}^{t}\right)\right\}}_{t=1\cdots M_{\bar{F}}}$ using Equation (19), where $M_{\bar{F}}$ is the number of elements in $S_{\bar{F}}$ and $S_{\bar{P}}$.

**Figure 6:**
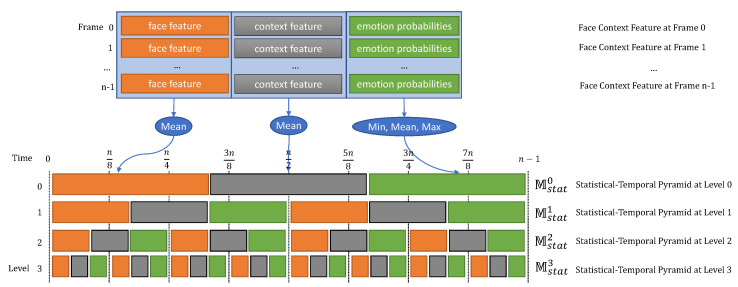
Context Temporal-Pyramid Features.

Then, the temporal-pyramid block $M_{stp}$ exploits the long-term temporal dependencies during all time steps through a temporal pyramid scheme. It consists of statistical aggregation $M_{stat}^{k=l_{1},l_{2},\cdots ,l_{P}}$ models where every statistical aggregation $M_{stat}^{k}$ transforms the face context features $\left\{Y_{fac\_ctx}^{t}\right\}$ into temporal pyramid features at level *k*, as shown in Figure 6.

The $M_{stat}^{k}$ model divides all time steps into k time step sub-sequences $TS_{j}^{k}={\left[\frac{\left(j-1\right)n}{k},\frac{j.n}{k}\right)}_{j=1\cdots k}$ where $n=S_{\bar{F}}$ is the number of faces and persons in $S_{\bar{F}}$ and $S_{\bar{P}}$. The face context features ${\left\{Y_{face\_ctx}^{i}\right\}}_{i\in TS_{j}^{k}}$ in every time step sub-sequence *j* are transformed into the temporal pyramid feature $V_{j}^{k}$ using the operator mean for face and context features and min, mean, and max for face probabilities, as follows:

(26) $$\begin{array}{c} \begin{array}{cc} V_{k,facef}^{j} & =mean_{i\in TS_{j}^{k}}\left\{Y_{facef}^{i}\right\}\\ V_{k,ctx}^{j} & =mean_{i\in TS_{j}^{k}}\left\{Y_{ctx}^{i}\right\}\\ V_{k,facep}^{j} & =concat\left(\min_{i\in TS_{j}^{k}}\left\{Y_{facep}^{i}\right\},mean_{i\in TS_{j}^{k}}\left\{Y_{facep}^{i}\right\},\max_{i\in TS_{j}^{k}}\left\{Y_{facep}^{i}\right\}\right) \end{array} \end{array}$$

where the mean, max, and min operators are used to create an aggregate of the mean, max, and min from the vector values and the concat operator combines all values in a vector. The correlation between $\left(V_{k,facef}^{j},V_{k,ctx}^{j}\right)$ and $V_{k,facep}^{j}$ is exploited at every time step sub-sequence *j* in pyramid level *k*, which helps our model learn the long-term temporal dependencies.

The temporal pyramid feature $V_{j}^{k}$ is a combination of $V_{k,facef}^{j}$, $V_{k,ctx}^{j}$, and $V_{k,facep}^{j}$ as follows:

(27) $$\begin{array}{c} \begin{array}{cc} V_{k}^{j} & =concat\left(V_{k,facef}^{j},V_{k,ctx}^{j},V_{k,facep}^{j}\right) \end{array} \end{array}$$

Finally, the temporal-pyramid block $M_{stp}$ incorporates $M_{stat}^{k}$ models in pyramid levels $k=\left\{l_{1},l_{2},\ldots ,l_{P}\right\},l_{i}\in \left[0\cdots 3\right]$ to produce the context temporal-pyramid feature $Y_{stp}$ as follows:

(28) $$\begin{array}{c} \begin{array}{cc} Y_{stp} & =concat_{k\in \left\{l_{1},l_{2},\cdots ,l_{P}\right\}}M_{stat}^{k}\left({\left\{\left(Y_{facep}^{t},Y_{facef}^{t},Y_{ctx}^{t}\right)\right\}}_{t=1\cdots M_{\bar{F}}}\right) \end{array} \end{array}$$

From there, we use the classification block $M_{clas}$, the architecture of which is similar to that of $M_{clas}$ in the context spatiotemporal model for emotion classification to produce emotion probabilities, as shown in Equation (24). We also apply categorical cross-entropy loss to train the model, as shown in Equation (25).

## 5. Best Selection Ensemble

The main idea of an ensemble method is to identify the best combination of the given models to solve the same tasks. The main advantage of ensemble methods is that they effectively use the large margin classifiers to reduce variance error and bias error [51].

We propose a **best selection ensemble** method to combine multi-modality information to address the bias error problem. Our method applies the heuristic principle to find the best combination of the given models at every selection step. We search all model combinations with the given first model and keep the shortest combination to prevent over-fitting.

First, it is assumed that the outputs of the ${\left\{M_{k}\right\}}_{k=1\cdots K}$ models are prediction emotion probability vectors ${\left\{{\hat{Y}}_{k}\right\}}_{k=1\cdots K}$ defined as follows:

(29) $$\begin{array}{c} \begin{array}{cc} {\hat{Y}}_{k} & ={\left\{{\hat{y}}_{k,i}\right\}}_{i\in \left[1,N_{E}\right]},\sum_{i\in \left[1,N_{E}\right]}{\hat{y}}_{k,i}=1 \end{array} \end{array}$$

where *K* and $N_{E}=7$ are the number of models and emotion labels, respectively. The average fusion $F_{avg}$ of ${\left\{M_{k}\right\}}_{k=1\cdots K}$ is calculated as follows:

(30) $$F_{avg}\left(\left\{M_{k}\right\}\right)={\left\{\frac{\sum_{k=1}^{K}{\hat{y}}_{k,i}}{K}\right\}}_{i\in \left[1,N_{E}\right]}$$

The multi-modal score is calculated based on the accuracy metric between the fusion result and the ground truth, as follows:

(31) $$\begin{array}{c} \begin{array}{cc} {\mathrm{Score}}_{acc}\left(\left\{M_{k}\right\}\right) & =acc\left(F_{avg}\left(\left\{M_{k}\right\}\right),Y_{gt}\right) \end{array} \end{array}$$

where the acc operator is used to calculate the accuracy of the prediction compared to ground truth.

Without loss of generality, we assume that ${\left\{M_{k}\right\}}_{k=1\cdots K}$ is sorted in descending accuracy where ${\mathrm{Score}}_{acc}\left(M_{i}\right)>{\mathrm{Score}}_{acc}\left(M_{j}\right)$ if i<j.

Let Select be the model-combination set. Initially, Select is empty. We sequentially choose the first model $M_{s_{1}}$ from left to right in ${\left\{M_{k}\right\}}_{k=1\cdots K}$ and attempt to find the optimal list of model selections corresponding to the given model $M_{s_{1}}$.

Let $Open={\left\{M_{k}\right\}}_{k=1\cdots K}\backslash \left\{M_{s_{1}}\right\}$ be the open list of models that can be selected for processing. $Close=\left\{M_{s_{1}}\right\}$ is then the closed list of the selected models.

At step *l*, it is assumed that $Open={\left\{M_{k}\right\}}_{k=1\cdots K}\backslash \left\{M_{s_{j=1\cdots l}}\right\}$ and $Close=\left\{M_{s_{j=1\cdots l}}\right\}$. We select the first model $M_{v}$ from left to right in Open such that the following is satisfied:

(32) $$\begin{array}{c} \begin{array}{cc} F_{avg}\left(Closed⋃\left\{M_{v}\right\}\right) & >F_{avg}\left(Closed\right)\\ \left|Closed⋃\left\{M_{v}\right\}\right|<=T \end{array} \end{array}$$

where T=5 is the threshold of the number of models in Closed (determined experimentally).

If a model $M_{v}$ cannot be found, we stop at this step and update the Select list as follows:

(33) $$\begin{array}{c} \begin{array}{c} Select=Select⋃\left\{Closed\right\} \end{array} \end{array}$$

We then repeat the process to select the first model in the next position. Finally, we choose the model combination in Select with the highest accuracy and smallest number of models.

## 6. Experiments and Discussion

### 6.1. Datasets

#### 6.1.1. Image-Based Emotion Recognition in the Wild

In this work, we chose suitable datasets for training of the face feature extraction model. The datasets must deal with the in-the-wild environments where there are many unconstrained conditions, such as occlusion, poses, illumination, etc. AffectNet [16] and RAF-DB [15] are by far the largest datasets satisfying the above criteria. The images in the datasets are collected from the Internet based on emotion-related keywords. Emotion labels are annotated by experts to guarantee reliability.

**Figure 7:**
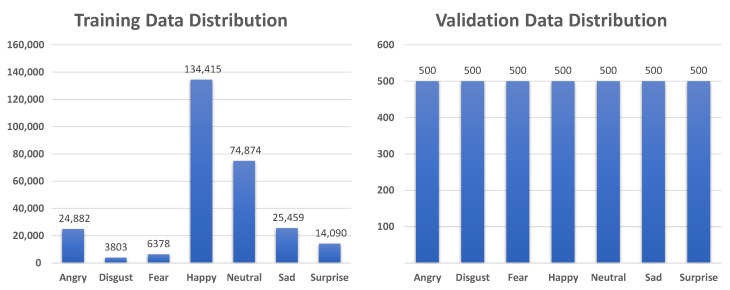
Data distribution for training and validation on AffectNet dataset [16].

AffectNet [16] contains two data groups, manual and automatic groups, with more than 1,000,000 images that are labeled with 10 emotion categories as well as dimensional emotion (valence and arousal). We used only images in the manual group belonging to seven basic emotion categories (anger, disgust, fear, happiness, neutrality, sadness, and surprise). Thus, we used 283,901 images for training and 3500 images for validation. The data distributions of in the training and validation sets are shown in Figure 7.

**Figure 8:**
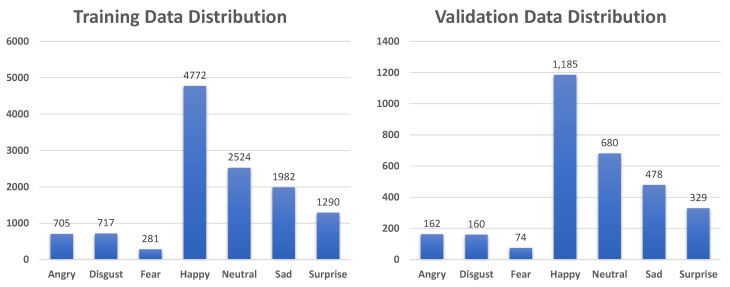
Data distribution for training and validation on RAF-DB dataset [15].

The RAF-DB dataset [15] consists of about 30,000 facial images in the basic and compound emotion groups which were taken under the in-the-wild conditions with illumination changes, uncontrolled poses, and occlusion. In this study, we chose 12,271 images for training and 3068 images for validation, all of which were from the basic emotion group. The data distributions of the training and validation sets are shown in Figure 8.

#### 6.1.2. Video-Based Emotion Recognition in the Wild

For facial emotion recognition in video clips, we used the AFEW dataset [33] to evaluate our study. The video clips in the dataset are collected from movies and TV shows under uncontrolled environments in terms of occlusion, illumination, and head poses. Each video clip was chosen based on its label, which contains emotion-related keywords corresponding to the emotion illustrated by the main subject. Use of this dataset helped us to address the problem of temporal facial expressions in the wild.

**Figure 9:**
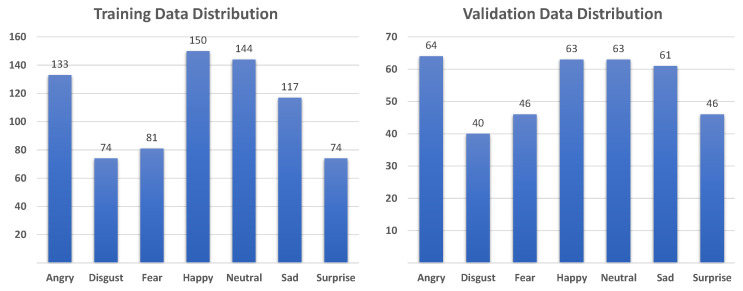
Data distribution for training and validation on AFEW dataset [33].

From the AFEW dataset, we used 773 video clips for training and 383 video clips for validation with labels corresponding to the seven basic emotion categories (anger, disgust, fear, happiness, neutrality, sadness, and surprise). The distribution of this dataset is shown in Figure 9.

Table 1 shows the datasets used for in image and video emotion recognition in this study:

### 6.2. Environmental Setup, Evaluation Metrics, and Experimental Setup

**Environment**. We used Python 3.7 with Tensorflow 2.1 and Keras to develop our program. Our experiments were conducted on a Desktop PC with Intel Core I7 8700, 64 GB RAM and two Nvidia GeForce GTX 1080 Ti graphic cards with 11 GB memory.

**Evaluation Metrics**. We used accuracy (Acc.) and $F_{1}$ score as the quantitative measurements in this study. We also used the average $Mean_{Acc.}$ and standard deviation $Std_{Acc.}$ of the accuracy values on the main diagonal of the normalized confusion matrix $M_{norm}$ to evaluate the performance results, as in [15]. These metrics are calculated as follows:

(34) $$\begin{array}{c} \begin{array}{cc} Accuracy & =\frac{TP+TN}{TP+FP+TN+FN}\\ F_{1} & =2\frac{Precision.Recall}{Precision+Recall}\\ Mean_{Acc.} & =\frac{\sum_{i=1}^{n}g_{i,i}}{n}\\ Std_{Acc.} & =\sqrt{\frac{\sum_{i=1}^{n}{\left(g_{i,i}-Mean_{Acc.}\right)}^{2}}{n}} \end{array} \end{array}$$

where $g_{i,i}\in diag\left(M_{norm}\right)$ is the *i*th diagonal value of the normalized confusion matrix $M_{norm}$, *n* is the size of $M_{norm}$, and TP, TN, FP, and FN, respectively, are true positive, true negative, false positive, and false negative. The precision is the ratio of correctly predicted positive samples to all predicted positive samples. The recall is the ratio of correctly positive prediction to all true samples. They are calculated as follows:

(35) $$\begin{array}{c} \begin{array}{c} Precision=\frac{TP}{TP+FP}\\ Recall=\frac{TP}{TP+FN} \end{array} \end{array}$$

The accuracy metric measures the ratio of correctly predicted samples to all samples; it ranges from 0 (worst) to 1 (best). It allows us to assess the performance of our model given that the data distribution is almost symmetric.

$F_{1}$ score can be used to more precisely evaluate the model in the case of an uneven class distribution, as it takes both FP and FN into account. $F_{1}$ score is a weighted average of precision and recall and ranges from 0 (worst) to 1 (best). In this study, due to the multi-class classification problem, we report the $F_{1}$ score as the weighted average $F_{1}$ score of each emotion label with weighting based on the number of labels.

Moreover, we also used $Mean_{Acc.}$ and $Std_{Acc.}$ to consider emotion evaluation under in-the-wild conditions with an imbalanced class distribution. This can be done in place of the accuracy metric, which is sensitive to bias under an uneven class distribution.

**Experimental Setup**. In this study, we conducted four experiments corresponding to: (1) the face and context feature extraction models; (2) the context spatiotemporal models; (3) the context temporal-pyramid model; and (4) the ensemble methods. Finally, we compared our results to related works on the AFEW dataset for video emotion recognition.

### 6.3. Experiments on Face and Context Feature Extraction Models

**Overview**. We used six conventional architectures to build a face feature extraction model to integrate into the facial emotion recognition models for video clips shown in Table 2. They consisted of Resnet 50 [18], Senet 50 [44], Densenet 201 [47], Nasnet mobile [46], Xception [45], and Inception Resnet [48]. Besides training from scratch, weights pre-trained on VGG-Face 2 [41], VGG-Face 1 [49], and ImageNet [50] were also used for transfer learning to leverage the knowledge from these huge facial and visual object datasets. For the context feature extraction model, we used the VGG16 model [17] with weights pre-trained on ImageNet [50] to extract the context feature around the person region.

**Training Details**. We first trained the models on the AffectNet dataset. We then fine-tuned the models on the RAF-DB dataset. Because the training and testing distributions differed, we applied a sampling technique to ensure that every emotion label in every batch had the same number of elements. Every image was resized to 224×224 and data augmentation was applied with random rotation, flip, center crop, and transition. The batch size was 8. The optimizer was Adam [52] with a learning rate of 0.001 and plateau reduction when training on the Affect-Net dataset. For fine-tuning on RAF-DB, we used SGD [53] with a learning rate within the range of 0.0004 to 0.0001 using the cosine annealing schedule.

**Results and Discussion**. Table 2 shows the performance measurements of the face feature extraction models on the validation sets of the AffectNet and RAF-DB datasets.

As shown in Table 2, the performance results on AffectNet could be separated into three distinct groups, which are, in descending order: Group 1 (Inception Resnet, ResNet 50, and Senet 50), Group 2 (Densenet 201 and Nasnet mobile), and Group 3 (Xception). Group 1 had three metrics greater than 61% with the highest accuracy value of 62.51%, $F_{1}$ score of 62.41% and $Mean_{Acc.}$ of 62.51% for the Inception Resnet model.

After fine-tuning on the RAF-DB dataset using the weights from pre-training on the AffectNet dataset, the ResNet 50 model achieved the best performance, with the accuracy of 87.22%, $F_{1}$ score of 87.38%, and $Mean_{Acc.}$ of 82.45%. $Mean_{Acc.}$ was 82.44% greater than that of the DLP-CNN baseline in the RAF-DB dataset (74.20%) [15]. Therefore, we chose to use this model as the face feature extraction model for video emotion recognition.

**Figure 10:**
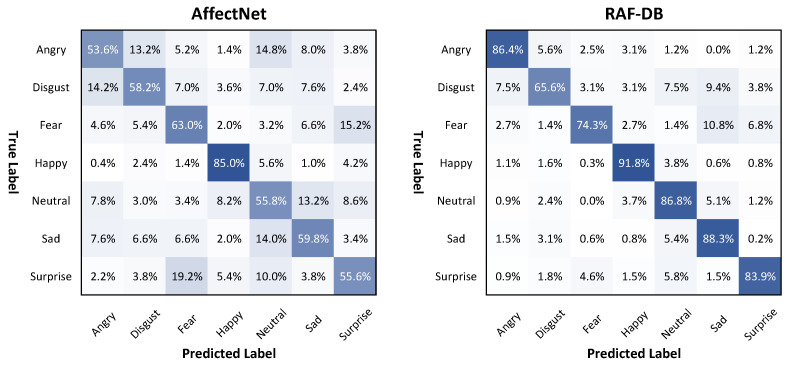
Confusion matrix of ResNet 50 model on the AffectNet and RAF-DB validation sets.

Figure 10 shows the confusion matrix of the ResNet 50 model on the validation sets of the AffectNet and RAF-DB datasets. For the results of the ResNet 50 model on AffectNet, the happiness emotion label achieved the highest accuracy of 85%, while the remaining emotion labels showed similar accuracies, ranging from 53.6% to 63%. After fine-tuning in the RAF-DB dataset, the accuracy of the images labeled neutrality, sadness, surprise, and anger were significantly enhanced from 83.9% to 88.3%, nearly reaching the accuracy of 91.8% for the happiness label. The disgust and fear categories showed the lowest accuracy. In addition, the values of $Mean_{Acc.}\pm Std$ on AffectNet and RAF-DB were 61.57%±10.78%, and 82.44%±9.20%, respectively.

### 6.4. Experiments on Spatiotemporal Models

**Overview**. The spatiotemporal models consist of four blocks, namely feature extraction block, LSTM block, 3DCNN block, and classification block that receives input from the face and person sequences. In this experiment, we built three different models from the spatiotemporal approach, as shown in Table 3.

Model 1, “Spatiotemporal Model + Fix-Feature,” used only the face sequence with the ResNet 50 face feature extraction model. The ResNet 50 model used weights that were pre-trained on the AffectNet and RAF-DB datasets, as discussed above. Moreover, all layers of the ResNet 50 model were frozen. Thus, the face feature extraction model was not fine-tuned during video-based emotion recognition training. Model 2, “Spatiotemporal Model + NonFix-Feature,” was different from the first model in that only three blocks of the ResNet 50 model were frozen, and the feature block of the ResNet 50 model was fine-tuned. Model 3, “Spatiotemporal Model + NonFix-Feature + Context,” expanded the context feature of Model 2 using input from both face and person sequences and used the pre-trained weights from the VGG16 model on ImageNet for context feature extraction.

**Training Details**. We trained our models on the AFEW dataset. During video batch sampling, every emotion label appeared with the same frequency to overcome the uneven class distribution and differences in distribution between the training and validation sets. We randomly extracted 32 frames per video clip in the training phase. For the validation phase, we averaged five predictions per clip by randomly extracting 32 frames. For data augmentation, we transformed the whole face and person sequence by resizing to 224 × 224, applying random horizontal flip, spatial rotation $\pm 15^{\circ }$, and scaling ±20%. Training was done using SGD optimizer with early stopping at 40 epochs, an initial learning rate of 0.0004, and a reduction in the learning rate on the plateau.

**Result Discussion**. Table 3 illustrates the performance results of the spatiotemporal models on the validation set of the AFEW dataset.

Model 1, with fixed face features due to frozen face feature extraction, obtained an accuracy of 51.70%, $F_{1}$ score of 54.17%, and $Mean_{Acc.}$ of 46.51%. Through fine-tuning on the feature block of the ResNet 50 model, Model 2showed an enhancement of accuracy by 0.52%, $F_{1}$ score by 2.09%, and $Mean_{Acc.}$ by 0.82%. Due to use of the context with the person region, Model 3 showed significant increases of 1.82%, 2.52%, and 1.65% for the accuracy, $F_{1}$ score, and $Mean_{Acc.}$, respectively. Model 3 also showed the highest accuracy of 54.05%, $F_{1}$ score of 50.78%, and $Mean_{Acc.}\pm Std$ of 48.98%±32.28% among all the spatiotemporal models.

**Figure 11:**
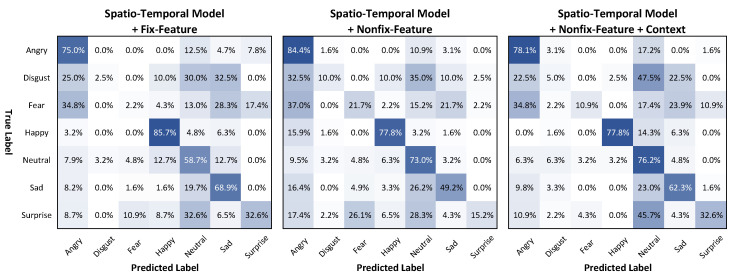
Confusion matrix of Models 1–3 (spatiotemporal approach) on the AFEW validation set.

Figure 11 shows the confusion matrix among the three models using the spatiotemporal approach. By fine-tuning the feature block of the face feature extraction model, Model 2 obtained an accuracy of 73% in the neutrality emotion label, compared to 58.7% for Model 1. Furthermore, Model 3, which took context into account, showed an enhancement of the accuracy of the sadness and surprise emotion labels, with accuracies of 62.3%, and 32.6%, respectively. These figures represent increases of 13.1% and 17.2% for the two emotion labels compared to the second approach. Moreover, Model 3 showed $Mean_{Acc.}\pm Std$ of 48.98%±32.28%, which is greater than the 47.33%±31.73% of Model 2, and 46.51%±34.38% of Model 1.

### 6.5. Experiments on Temporal-Pyramid Models

**Overview**. For the temporal-pyramid model, we performed an ablation study on the context and scale factors, as shown in Table 4. For the context factor, Models 4–6 without context used only the ResNet 50 face feature extraction model, while Models 7–9 with context combined the face and context features from face and person sequences. When shown a face frame, a model without context produced one vector with a length of 2048 for the face feature and 21 probability outputs corresponding to the seven emotion labels and three statistical operators (min, mean, and max). The context feature vector from the VGG 16 model using pre-trained weights form ImageNet had a length of 2048. Therefore, the models without/with context had lengths of 2069/4117 per frame.

For the level factor, we conducted experiments on three groups of levels, {3}, {4}, and {0,1,2,3}. At level k, all processing frames are divided into $2^{k}$ sub-sequences and all sub-sequences are combined in the same interval by the mean operator in the face and context features and three operators (min, mean, and max) in the emotion probability outputs. For example, for level group {0,1,2,3}, we divided all face and context frames in a video clip into 1, 2, 4, and 8 sub-sequences at Levels 0, 1, 2, and 3, respectively. In total, 15 sub-sequences were used to capture the emotion based on statistical information from whole frames or small chunks of frames with various lengths. Therefore, the length of the temporal-pyramid features without and with context is 15 * 2069 = 31,035 and 15 * 4117 = 61,755, respectively.

**Training Details**. In the training phase, we created temporal-pyramid features at level groups {3}, {4}, and {0,1,2,3} with and without context using the face feature model and context feature model with pre-trained weights in the Resnet 50 model from AffectNet and RAF-DB, and pre-trained weights for the VGG-16 model from ImageNet. For every level group, we used data augmentation to process 10 instances in every video clip. Data augmentation was applied to all frames with the same transformations: resizing to 224 × 224, random horizontal flip, scaling, and rotation. When sampling to get a minibatch, we randomly chose eight video clips with one of ten instances in data augmentation for every video clip, where the results satisfied the balance between emotion labels in a minibatch. We used the same training configuration as used in the training phase of the spatiotemporal models with the SGD optimizer, an initial learning rate 0.0004, and learning rate reduction on the plateau.

**Results and Discussion**. Table 4 depicts the experimental results of the temporal-pyramid models with adjustment of context and level factors.

For the level factor, Models 4–6, respectively, were set to level groups {3}, {4}, and {0,1,2,3} without context. The performance results of the three models were the same, with an accuracy of 55.87%. However, Model 6, with many level factors, gave better results in terms of $F_{1}$ score and $Mean_{Acc.}$ (54.06% and 51.85%, respectively, compared to 52.76% and 51.21% and 52.51%, and 51.23% for Models 4 and 5, respectively). Similarly, Model 9, using many level factors, also showed an $F_{1}$ score and $Mean_{Acc.}$ of 56.50% and 54.25%, which were superior to the results of Models 7 and 8. Therefore, the level factor affected the $F_{1}$ score and $Mean_{Acc.}$.

For the context factor, Models 7–9. respectively, increased accuracy, $F_{1}$ score, and $Mean_{Acc.}$ by 0.26%, 1.86%, and 1.15%; 0.52%, 1.49%, and 0.94%; and 0.78%, 2.44%, and 2.41% over the corresponding values of Models 4–6. In the same level group, the context factors helped Models 7–9 provide better results than Models 4–6, respectively. Moreover, Model 9, with many level factors, showed a significant increase in $F_{1}$ score and $Mean_{Acc.}$, as it had the highest values of 56.50% and 54.25%, respectively.

**Figure 12:**
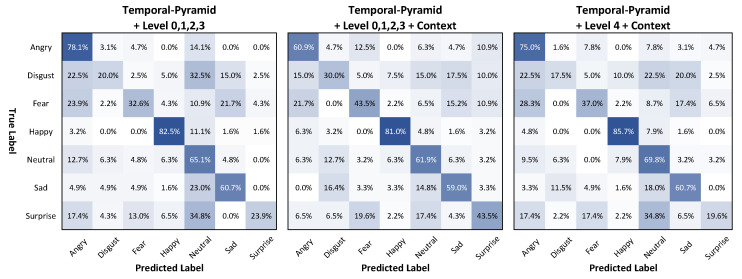
Confusion matrices of Models 6, 9, and 8 (from left to right), which use the temporal-pyramid approach on the AFEW validation set.

Figure 12 shows the confusion matrices of Models 6, 9, and 8. For the same level group {0,1,2,3}, Model 9, with context, showed an enhancement in the accuracy of the difficult emotion labels, disgust, fear, and surprise, by 30.0%, 43.5%, and 43.5%, respectively, compared to 20.0%, 32.6%, and 23.9% for Model 6. The $Mean_{Acc.}\pm Std$ of Model 9 was 54.25%±16.63%, which is greater than the 51.84%±25.98% of Model 6 (without context) and 52.18%±27.47% of Model 8 (with only one level {4}).

### 6.6. Experiments on Best Selection Ensemble

**Overview**. We conducted ensemble experiments through three approaches to exploit the complementary nature and redundancy among the models, as shown in Table 5. We first used the average fusion method, which combines the seven emotion probability outputs of all models with an average operator. The second approach was the multi-modal joint late-fusion method [10]. In this approach, we divided all models into two groups, spatiotemporal (Models 1–3) and temporal-pyramid (Models 4–9) groups. This method used the average operator to merge all probability outputs of the emotion models in the same group, called the probability-merged layer, followed by a dense layer, and a softmax layer for classification into the seven emotion categories. The role of each group’s outputs guarantees the accuracy of each branch. In addition, the model had a joint branch to merge the probability-merged layers of the two groups with a concatenation operator to give the emotion outputs.

The last approach was the best selection ensemble method. It chooses one of the models as the first element and then repeats the process by adding one of the remaining models using the average operator on the probability outputs with the previous models to help current combination increase. The process ends when there are no additional unused models to help increase the accuracy of the model combination or all models are selected.

**Results and Discussion**. The results of our experiments on the average fusion, multi-model joint late fusion, and best selection ensembles are shown in Table 5.

The best selection method showed the highest accuracy and $F_{1}$ score of 59.79% and 58.48%, respectively, representing significant increase in accuracy and $F_{1}$ score of 2.09% and 3.48% and 1.3% and 1.08% compared to the average fusion method and multi-modal joint late-fusion method, respectively. The combination models in the best selection method that gave the best scores were Models 3, 6, 7, and 9.

**Figure 13:**
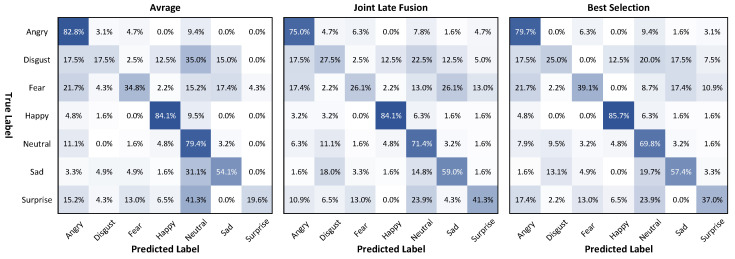
Confusion matrices of the average fusion method, multi-modal join late-fusion method and best selection ensemble method on the AFEW validation set.

The confusion matrix in the best selection method shown in Figure 13 gave the highest $Mean_{Acc}$ 56.24% with the smallest $Std_{Acc.}$ of 23.26% compared to the average fusion method and multi-modal join late-fusion method. Moreover, this method showed an improvement in performance for the more difficult emotion labels: disgust, 25.0%; fear, 39.1%; and sadness, 37.0%.

### 6.7. Discussion and Comparison with Related Works

**Figure 14:**
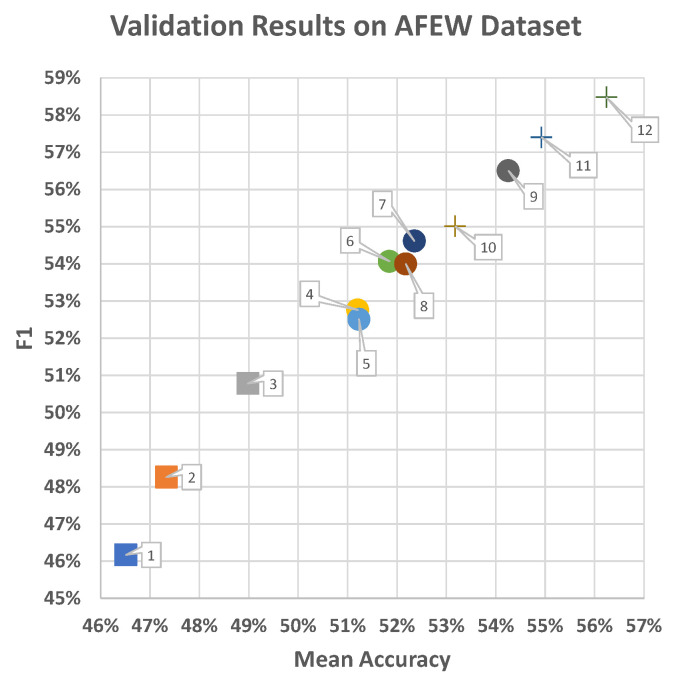
Results of our proposed models on the AFEW validation set. The rectangle data points represent spatiotemporal approaches, with context (Model 3) and without context (Models 1 and 2). The circular data points represent models based on temporal-pyramid approaches, which consist of two groups: without context (Models 4–6) and with context (Models 7–9) with level groups of {3}, {4}, {0,1,2,3}, respectively, in each group. Finally, the “plus sign” data points represent the average fusion method (Model 10), multi-modal joint late-fusion method (Model 11), and best selection method (Model 12).

**Discussion**. Figure 14 presents the results of the three experiments on the AFEW validation set. First, the context factor played an important role in enhancing the performance of spatiotemporal Model 3 compared to Models 1 and 2 using the same approach, as well as temporal-pyramid Models 7–9 compared to the corresponding Models 4–6. This finding confirms that context is key to interpretation facial expression to access the emotional state of a person [54], especially, in cases in which the facial region is small and blurry.

Second, use of multi-level factors {0,1,2,3} in temporal-pyramid models provided more robust features than were seen in the models using only a single level ({3} and {4}). For instance, Model 6 gave better results than Models 4 and 5. Similarly, the performance of Model 9 was better than that of Models 7 and 8. This shows that division of time periods in facial expression representation in a hierarchical structure creates robust features to capture human emotions under in-the-wild conditions, such as unclear temporal border and multiple apexes from spontaneous expressions.

Finally, when integrating multiple-modalities, the best selection ensemble method achieved better results than average fusion method, and multi-modal joint late-fusion method.

The main advantage of our ensemble method is that it allows the identification of the best combination of a large number of models through a multi-modal approach as well as derivation of instances from many training times. We were able to expand the average operator through use of other operators, such as skew, min, max, and median, as well as by combining many operators. In this study, the average and median operator were more useful than the others.

**Comparison with related works**. The accuracy measurements of our proposed methods and related methods on the AFEW validation set are shown in Table 6.

Our spatiotemporal method outperforms other recently reported methods using the same approach, by around 0.14% compared with Li et al. [63]. Recently, Kumar et al. [66] used multi-level attention with an unsupervised approach by iterative training between student and teacher models. Their method showed a highest accuracy of 55.17%, which is lower than that of our temporal-pyramid method, 56.66%. To compare the fusion and ensemble methods, we searched for related studies that used multiple-modalities using visual and geometric information of facial expressions. Our ensemble method achieved the highest accuracy of 59.79%, which is better than that shown in related studies, where the highest reported accuracy was 57.43% by Fan et al. [61].

## 7. Conclusions

In this study, we built an emotion recognition system to track the main face and recognize its facial expression in a video clip. We propose a face-person tracking and voting module to help our system detect the main face and person in a video clip for emotion recognition. Our tracking algorithm is based on a tracking-by-detection scheme with robust appearance observations to suggest facial and human regions, while the voting module uses relevant information about frequency of occurrences, size, and face detection probability to determine a main human and face sequence. In the next step, our emotion recognition models detects facial expressions through two main approaches, the spatiotemporal approach and the temporal-pyramid approach. Finally, the best selection ensemble method selects the best combination of models from among many training models to predict facial expression in a video clip. Compared to previous results on the AFEW dataset, our work shows improvement in every domain.

In the spatiotemporal models, we use 2D CNN facial and context blocks followed by an LSTM block and 3D CNN block to exploit the spatiotemporal coherence of facial and context features and facial emotion probabilities. The context factor is a significant key that increases the the performance of our model from 52.22% to 54.05%. Moreover, we achieved an accuracy that is better than that reported by related studies on the AFEW validation set.

For the temporal-pyramid models, we apply data augmentation on facial and context regions and extracted facial and person features and face emotion probabilities from every frame of the video clip. Using temporal-pyramid strategies, we created robust hierarchical features to feed into a simple neural network for classification of facial expression. Our method exploits the high correlation of features in the temporal domain. Due to the improvements mentioned above, we achieved an accuracy of 56.66% on the validation set, which is better than the accuracies of related studies using a single model with the same approach.

Finally, we propose a best selection ensemble to select a suitable combination of models from a large number of model instances during training with tuning of hyper-parameters, adjustment of levels, and configuration of context factor. Our ensemble method achieved an accuracy of 59.79%, which is better than that of the average fusion and multi-modal joint late-fusion method as well as related studies on the AFEW validation set.

In the further works, we will apply a multi-level attention mechanism to highlight the spatiotemporal correlations between emotion features over time. In addition, we use a graph convolution network to express movement of facial action units, which helps our system to better classify human expression.

## Figures and Tables

**Table 1:** Image and video emotion recognition datasets.

Emotion	AffectNet [16]		RAF-DB [15]		AFEW [33]	
	Training	Validation	Training	Validation	Training	Validation
Angry	24,882	500	705	162	133	64
Disgust	3803	500	717	160	74	40
Fear	6378	500	281	74	81	46
Happy	134,415	500	4772	1185	150	63
Neutral	74,874	500	2524	680	144	63
Sad	25,459	500	1982	478	117	61
Surprise	14,090	500	1290	329	74	46
Total	283,901	3500	12,271	3068	773	383

**Table 2:** Performance of face feature extraction models on the AffectNet and RAF-DB validation sets.

No	Model	Pre-Train Weight	Affectnet [16]			RAF-DB [15]		
			Acc.	$F_{1}$	${\mathrm{Mean}}_{\mathrm{Acc}}\pm \mathrm{Std}$	Acc.	$F_{1}$	${\mathrm{Mean}}_{\mathrm{Acc}}\pm \mathrm{Std}$
1	ResNet 50 [18]	VGGFace2 [41]	61.57%	61.46%	61.57%±10.79%	**87.22**%	**87.38**%	**82.45**% ± **09.20**%
2	Senet 50 [44]	VGGFace 1 [49]	61.51%	61.50%	61.51%±10.40%	83.64%	83.81%	76.96%±11.12%
3	Nasnet mobile [46]	ImageNet [50]	59.20%	58.88%	59.20%±13.95%	80.74%	81.01%	74.05%±12.44%
4	Densenet 201 [47]	ImageNet [50]	59.31%	58.91%	59.31%±14.12%	83.08%	83.23%	76.94%±11.31%
5	Inception Resnet [48]	Scratch	62.51%	62.41%	62.51%±09.63%	81.23%	81.79%	77.08%±08.10%
6	Xception [45]	Scratch	56.26%	56.38%	56.26%±11.18%	80.90%	81.03%	74.71%±14.28%

**Table 3:** Performance results of the spatiotemporal models on the AFEW validation set.

No	Method	Context	Feature	Acc.	$F_{1}$	${\mathrm{Mean}}_{\mathrm{Acc}.}\pm \mathrm{Std}$
1	Spatiotemporal Model + Fix-Feature		Fix	51.70%	46.17%	46.51%±34.38%
2	Spatiotemporal Model + Nonfix-Feature		Nonfix	52.22%	48.26%	47.33%±31.73%
3	Spatiotemporal Model + Nonfix-Feature + Context	✔	Nonfix	**54.05**%	**50.78**%	**48.98**% ± **32.28**%

**Table 4:** Performance results of the temporal-pyramid models on the AFEW validation set.

No	Method	Context	Level	Acc.	$F_{1}$	${\mathrm{Mean}}_{\mathrm{Acc}.}\pm \mathrm{Std}$
4	Temporal-Pyramid Model + Level 3		3	55.87%	52.76%	51.21%±29.87%
5	Temporal-Pyramid Model + Level 4		4	55.87%	52.51%	51.23%±30.12%
6	Temporal-Pyramid Model + Level 0,1,2,3		0,1,2,3	**55.87**%	**54.06**%	**51.85**% ± **25.98**%
7	Temporal-Pyramid Model + Level 3 + Context	✔	3	56.14%	54.61%	52.35%±25.53%
8	Temporal-Pyramid Model + Level 4 + Context	✔	4	56.40%	53.99%	52.18%±27.47%
9	Temporal-Pyramid Model + Level 0,1,2,3 + Context	✔	0,1,2,3	**56.66**%	**56.50**%	**54.25**% ± **16.63**%

**Table 5:** Validation results of the ensemble experiments on the AFEW validation set.

No	Method	Acc.	$F_{1}$	${\mathrm{Mean}}_{\mathrm{Acc}.}\pm \mathrm{Std}$
10	Average Fusion	57.70%	55.00%	53.18%±29.62%
11	Multi-modal Joint Late-Fusion [10]	58.49%	57.40%	54.92%±23.50%
12	Best Selection Ensemble	**59.79%**	**58.48**%	**56.24**% ± **23.26**%

**Table 6:** Performance comparison with related studies on AFEW validation set.

Authors	Method	Approach	Modality	Year	Accuracy
Fan et al. [55]	CNN + LSTM	Spatiotemporal (2D + T)	Visual	2016	45.43%
	C3D	Spatiotemporal (3D)	Visual		39.69%
Yan et al. [56]	Trajectory Features + SVM	Geometry	Geometry	2016	37.37%
	CNN Features + Bi-directional RNN	Spatiotemporal (2D+T)	Visual		44.46%
	Fusion	Fusion	Visual + Geometry		49.22%
Vielzeuf et al. [57]	VGG-LSTM	Spatiotemporal (2D+T)	Visual	2017	48.60%
	LSTM C3D	Spatiotemporal (3D)	Visual		43.20%
	ModDrop Fusion	Fusion	Visual		52.20%
Hu et al. [58]	Face Features + Supervised Scoring Ensemble	Frame-Level	Visual	2017	44.67%
Knyazev et al. [28]	Face Features + STAT (min,std,mean) + SVM	Frame-Level	Visual	2017	53.00%
	Weighted Average Score	Fusion	Visual		55.10%
Kaya et al. [59]	CNN-FUN Features + Kernel ELMPLS	Spatiotemporal (3D)	Visual	2017	51.60%
Lu et al. [25]	VGG-Face + BLSTM	Spatiotemporal (2D+T)	Visual	2018	53.91%
	C3D	Spatiotemporal (3D)	Visual		39.36%
	Weighted Average Fusion	Fusion	Visual		56.05%
Liu et al. [26]	VGG16 FER2013 + LSTM	Spatiotemporal (2D+T)	Visual	2018	46.21%
	Face Features + STAT (min,std,mean) + SVM	Frame-Level	Visual		51.44%
	Landmark Euclidean Distance	Geometry	Geometry		39.95%
	Weighted Average Fusion	Fusion	Visual + Geometry		56.13%
Vielzeuf et al. [60]	Max Score Selection + Temporal Pooling	Frame-Level	Visual	2018	52.20%
Fan et al. [61]	Deeply-Supervised CNN (DSN)	Frame-Level	Visual	2018	48.04%
	Weighted Average Fusion	Fusion	Visual		57.43%
Duong et al. [62]	CNN Features + LSTM	Spatiotemporal (2D+T)	Visual	2019	49.30%
Li et al [63]	VGG-Face Features + Bi LSTM	Spatiotemporal (2D+T)	Visual	2019	53.91%
Meng et al. [64]	Frame Attention Networks (FAN)	Frame-Level	Visual	2019	51.18%
Lee et al. [65]	CAER-Net	Spatiotemporal (2D+T)	Visual	2019	51.68%
Kumar et al. [66]	Noisy Student Training + Multi-level attention	Frame-Level	Visual	2020	55.17%
**Our method**	**Spatiotemporal model**	Spatiotemporal (2D+T)	Visual		**54.05**%
	**Temporal-pyramid model**	Frame-Level	Visual		**56.66**%
	**Best Selection Ensemble**	Fusion	Visual		**59.79**%

## Data Availability

Not applicable.
